# mRNA Decay Factor AUF1 Binds the Internal Ribosomal Entry Site of Enterovirus 71 and Inhibits Virus Replication

**DOI:** 10.1371/journal.pone.0103827

**Published:** 2014-07-31

**Authors:** Jing-Yi Lin, Mei-Ling Li, Gary Brewer

**Affiliations:** 1 School of Medical Laboratory Science and Biotechnology, College of Medical Science and Technology, Taipei Medical University, Taipei, Taiwan; 2 Department of Biochemistry & Molecular Biology, Rutgers Robert Wood Johnson Medical School, Piscataway, New Jersey, United States of America; Centro de Biología Molecular Severo Ochoa (CSIC-UAM), Spain

## Abstract

AU-rich element binding factor 1 (AUF1) has a role in the replication cycles of different viruses. Here we demonstrate that AUF1 binds the internal ribosome entry site (IRES) of enterovirus 71 (EV71) and negatively regulates IRES-dependent translation. During EV71 infection, AUF1 accumulates in the cytoplasm where viral replication occurs, whereas AUF1 localizes predominantly in the nucleus in mock-infected cells. AUF1 knockdown in infected cells increases IRES activity and synthesis of viral proteins. Taken together, the results suggest that AUF1 interacts with the EV71 IRES to negatively regulate viral translation and replication.

## Introduction

Enterovirus 71 (EV71), a positive-stranded RNA virus of the *Picornaviridae* family, poses a persistent global health problem. EV71 presents most frequently as a child herpangina or exanthema, known as hand, foot and mouth disease (HFMD). However, acute EV71 infection is also associated with severe neurological complications with significant mortality. Children under five years old are particularly susceptible to the most severe forms of EV71-associated neurological complications, including aseptic meningitis, brainstem and/or cerebellar encephalitis, myocarditis, acute flaccid paralysis, and fatal pulmonary edema and hemorrhage [1]. Such presentations, as well as a poliomyelitis-like syndrome, have been observed during outbreaks in Taiwan, mainland China, Malaysia, Singapore, Western Australia, the United States and Europe [2]–[8].

During infection by poliovirus (PV), human rhinovirus (HRV), EV71, or coxsackievirus, the viral proteases 3C and 2A cleave cellular proteins, including the translation initiation factor eIF4G, causing rapid termination of host, cap-dependent translation [9]–[11]. IRES-mediated initiation of translation allows viral RNA translation while host cell translation is shut down during infection. IRES-dependent translation depends on both canonical translation initiation factors and IRES-specific trans-acting factors (ITAFs). Sweeney et al. [12] reconstituted type 1 IRES activity *in* *vitro* with three type 1 IRESs: poliovirus, EV71, and bovine enterovirus (BEV). Poliovirus, EV71, and BEV IRESs require eIF2, eIF3, eIF4A, eIF4G, eIF4B, eIF1A, and PCBP2 [poly(C) binding protein 2]. Initiation starts with binding of eIF4G/eIF4A and then recruitment of 43S complexes which strictly requires direct contact of eIF3 and eIF4G. The subsequent events can differ between IRESs, depending on the stability of stem-loop (SL)-VI. For the EV71 IRES, initiation events occur without inspection of SL-VI, implying that its AUG does not determine ribosomal attachment. ITAFs interact with various IRES segments to regulate their activities by affecting ribosome recruitment or by modifying IRES structure. Several proteins that modulate the EV71 IRES and function as ITAFs are known. These include polypyrimidine tract binding protein (PTB), poly(rC)-binding protein 1 (PCBP1), PCBP2, hnRNP K, hnRNP A1, far upstream binding protein 1 (FBP1), and FBP2 [13]–[17].

AU-rich elements (AREs) are well characterized mRNA-destabilizing elements in eukaryotes [18]. They reside within the 3′ untranslated regions (UTRs) of many mRNAs encoding cytokines, cell cycle regulators, signaling proteins, and oncoproteins. AUF1/hnRNP D is a family of ARE-binding proteins that controls degradation and/or translation of numerous ARE-mRNAs [19]. Alternative pre-mRNA splicing generates the four isoforms with apparent molecular masses of 37, 40, 42, and 45 kDa. The isoforms assemble with a complex of proteins, including heat shock proteins Hsc/Hsp70 and Hsp27, eIF4G, poly(A)-binding protein, and additional unknown proteins [20]–[22]. The AUF1 complex of proteins recruits messenger ribonuclease activities, and in some cases microRNAs, to degrade ARE-mRNAs [18]. AUF1 is also involved in the replication of some viruses such as hepatitis B, hepatitis C, Epstein-Barr, polio-, human rhino-, coxsackie, gammaherpes, Nipah, herpes viruses and HIV [23]–[31].

In this work we report AUF1 associates with the 5′UTR of EV71 and negatively regulates viral protein synthesis. The major site of AUF1 accumulation shifts from the nucleus to the cytoplasm upon EV71 infection of cells. An approach involving pull-down of protein-biotinylated RNA shows AUF1 interacts with SL-II in the 5′UTR. AUF1 knockdown promotes viral protein synthesis and increases virus titer indicating that AUF1 is a negative-acting ITAF of EV71.

## Materials and Methods

### Cells and virus

SF268 (human glioblastoma) cells [32] were cultured at 37°C in RPMI medium supplemented with 10% fetal calf serum (FCS) (Mediatech). RD (human embryonal rhabdomyosarcoma) [33] and Vero (African green monkey kidney) cells [34] were cultured at 37°C in Eagle’s minimum essential medium (MEM) supplemented with 10% FCS. EV71 (TW/2231/98) was propagated in RD cells. Cells were infected with EV71 at the specified multiplicity of infection (MOI) and then incubated at 37°C for 2 h for adsorption. Unbound virus was removed, and the cells were fed fresh medium. Media from infected cultures were harvested at the indicated times, and titers of EV71 were measured by plaque formation on Vero cells.

### Plasmid construction and in vitro transcription

The pT7-EV71 5′UTR was constructed as follows: the 5′UTR of EV71 was amplified by PCR from a full-length, infectious cDNA [35] using forward 5′-GCCGGTAATACGACTCACTATAGGGAGATTAAAACAGCCTGTGGGT and reverse 5′-CATGTTTGATTGTGTTGAGGGTCAAAAT primers that contain the T7 promoter. The PCR product was inserted into plasmid pCRII-TOPO by TA cloning (Life Technologies). The full-length EV71 5′UTR was also used in PCR reactions with appropriate primers to generate fragments containing various stem-loop regions. These also contain a T7 promoter for RNA synthesis. (Inclusive nucleotide numbers for each stem-loop construct are shown in the figure legend).

The T7 promoter–EV71-5′UTR fragment and stem-loop–containing fragments, flanked by EcoRI sites, were excised from pCRII-TOPO. RNAs were synthesized using the MEGAscript T7 kit (Life Technologies) according to the manufacturer’s protocol. Biotinylated RNA was synthesized in a 20 µl MEGAscript transcription reaction mixture by addition of 1.25 µl of 20 mM biotinylated UTP, Bio-16-UTP (Roche). Synthesized RNAs were purified using the RNeasy Mini Kit (Qiagen) and analyzed on 1% agarose gels.

The monocistronic reporter plasmids pEV71-5′UTR-FLuc and pRLuc were described previously; these contain a T7 promoter for synthesis of control monocistronic RNAs for transfection [13]. The bicistronic reporter plasmid was constructed as described previously [14]. Briefly plasmid pRHF-EV71-5′UTR, which contains the EV71 IRES inserted between Renilla and Firefly luciferase open reading frames, was constructed by ligating a NotI–EV71-5′UTR–NotI fragment into pRHF. The plasmid also contains a T7 promoter (downstream of the CMV promoter) to permit synthesis of RNA for transfection. pRHF-EV71-5′UTR was linearized by Drd I digestion and used as template for the synthesis of RLuc-EV71-5′UTR-FLuc RNA using the MAXIscript kit (Life Technologies) with inclusion of m^7^G(5′)ppp(5′)G to add cap to the 5′ end.

### Determination of EV71 IRES activity

SF268 cells were seeded in 12-well plates in antibiotic-free RPMI. Two hundred nanograms of RLuc-EV71-5′UTR-FLuc RNA was mixed with 5 µl SuperFect transfection reagent (Qiagen) in 400 µl MEM supplemented with 10% FCS for transfections following the manufacturer’s directions. Control monocistronic RNAs were transfected in separate reactions. IRES activity was determined 2 days after transfection by measuring Renilla luciferase (RLuc) and Firefly luciferase (FLuc) activities in a 20/20 luminometer (Turner Biosystems) using a dual-luciferase reporter assay system (Promega) according to the manufacturer’s instructions.

### Preparation of SF268 cell extracts

SF268 cells were grown in RPMI medium supplemented with 10% fetal bovine serum (Mediatech). Upon confluence, cells were pelleted, washed three times with cold PBS, resuspended in CHAPS buffer (10 mM Tris-HCl pH 7.4, 1 mM MgCl_2,_ 1 mM EGTA, 0.5% CHAPS, 10% glycerol, 0.1 mM PMSF, 5 mM 2-ME), and then incubated on ice for 30 min for swelling. Cells were lysed by centrifugation at 10,000×*g* for 10 min at 4°C, and the supernatants were collected for further analysis. Protein concentrations of cell extracts were determined using the Bio-Rad protein assay (Bio-Rad).

### Pull-down of protein-biotinylated RNA complexes using streptavidin beads

Reaction mixtures contained 200 µg of cell extract proteins and 3 µg of biotinylated EV71 5′-UTR RNA (or fragments thereof). The reaction mixture’s final volume was adjusted to 100 µl with RNA mobility shift buffer [5 mM HEPES (pH 7.1), 40 mM KCl, 0.1 mM EDTA, 2 mM MgCl_2_, 2 mM dithiothreitol, 1 U RNasin and 0.25 mg/ml heparin]. The mixtures were incubated for 15 min at 30°C and then added to 400 µl of Streptavidin MagneSphere Paramagnetic Particles (Promega) for binding at room temperature for 10 min. Beads, containing RNA-protein complexes, were washed five times with RNA mobility shift buffer lacking heparin. After the last wash, 15 µl of 6×SDS-PAGE sample buffer was added to the beads and the mixtures were incubated for 10 min at room temperature. The eluted proteins were boiled and fractionated by 10% SDS-PAGE. Specific proteins were detected by Western blot analyses as described below. Expression and purification of recombinant His_6_-p40^AUF1^ for competition assays was described previously [36].

### Western blot analysis

Cells were lysed in SDS sample buffer. Proteins were fractionated by 10% SDS-PAGE and transferred to nitrocellulose membranes by wet transfer. Membranes were blocked with phosphate-buffered saline (PBS) containing 5% low-fat dry milk (Blotto). Membranes were incubated with primary antibody and then washed with PBS, 0.2% Tween 20. Membranes were incubated with goat anti-mouse or anti-rabbit IgG conjugated to horseradish peroxidase (Promega). Reactions were developed using an enhanced chemiluminescence (ECL) kit (Thermo Scientific) and detected with X-ray film or the Kodak Image Station 4000R imaging system (Carestream). Primary antibodies were used at the following dilutions or concentrations: anti-AUF1 rabbit polyclonal, 115,000; anti-3C mouse polyclonal [37], 1750; anti-FBP1 goat polyclonal (Santa Cruz), 1200; anti-hnRNP A1 mouse monoclonal (Abcam), 1200; anti-hnRNP A2 mouse monoclonal (Abcam), 1200; anti-β actin rabbit polyclonal (Abcam), 15,000. To permit sequential detection of different proteins, antibodies were removed from membranes by washing them with OneMinute stripping buffer (GM Biosciences).

### Knockdown of AUF1 and hnRNP A1/A2

Two micrograms of plasmid expressing control or AUF1 shRNAs (pSilencer-U6-hygro/shCTRL or pSilencer-U6-hygro/shAUF1) [22] were mixed in 100 µl serum-free MEM and combined with 10 µl SuperFect reagent (Qiagen); this mixture was incubated at room temperature for 10 min before transfecting into cells. To reduce expression of hnRNP A1 and hnRNP A2, SF268 cells were seeded in 12-well plates in antibiotic-free media. One hundred nmol of each siRNA targeting hnRNP A1 and hnRNP A2 (ON-TARGETplus SMARTpool L-008221-00-0005 or L-011690-01-0005, Dharmacon) was transfected along with 3 µl Lipofectamine 2000 transfection reagent (Life Technologies) in 0.4 ml RPMI supplemented with 10% FCS following the manufacturer’s directions. To co-transfect shAUF1 and siRNAs targeting hnRNPs A1 and A2, Lipofectamine 2000 was used. Knockdown efficiency was monitored by Western blotting.

### Determination of viral RNA replication

SF268 cells were infected with EV71 at an MOI of 40. Cells were harvested at various time points, and total RNA was extracted from cells using an RNeasy minikit (Qiagen). Total RNA (500 ng) was reverse transcribed into cDNA with a TaqMan reverse transcription (RT) kit (Applied Biosystems) according to the manufacturer’s instructions. The resulting cDNA was analyzed by quantitative RT-PCR (qRT-PCR) using Power SYBR green PCR master mix (Applied Biosystems) and 15 pmol of each primer (for EV71 positive-strand RNA, 5′-CTGTAAATCAACGATCAATAGCAG, and 5′-GTAGTTGGTCGGGTAACGAAC; for β-actin, 5′-TGGCGCTTTTGACTCAGGAT and 5′-GGGATGTTTGCTCCAACCAA). Reactions were carried out using the Stratagene MX3005P thermocycler. Relative viral RNA levels were calculated based on standard curves.

### RNP-immunoprecipitation

Immunoprecipitations of endogenous protein-RNA complexes were used to assess association of AUF1 with EV71 RNA in infected cells. To pre-clear lysates in preparation for immunoprecipitation with AUF1 antibody, lysates of 6×10^7^ mock- or EV71-infected SF268 cells were incubated with rabbit non-immune serum (Sigma) for 45 min at 4°C, then magnetic Dynabeads coupled to protein A (Invitrogen) were added for 30 min at 4°C; beads were removed with a magnet. For immunoprecipitations, fresh beads were coated with anti-AUF1 or rabbit non-immune serum in NT-2 buffer [50 mM Tris-HCl (pH 7.4), 1 mM MgCl_2_, 150 mM NaCl and 0.05% Nonidet P-40] and washed. Pre-cleared cell lysates (1 mg protein) were incubated with 50 µl of coated beads in 200 µl of NT-2 buffer supplemented with 2.5 µ l of RNase Out (Invitrogen) and 2 µl of 100 mM DTT for 3.5 h at 4°C with constant rocking. Beads were washed eight times with ice-cold NT-2 buffer and two times with NT-2 buffer supplemented with 0.5 M urea. Proteins were digested with proteinase K (Promega), and RNAs were purified by phenol-chloroform extraction and ethanol precipitation. RNAs were then analyzed by Northern blotting as described [38]. Briefly, RNA from each sample was denatured by the addition of formaldehyde and formamide (final concentrations of 2.2 M and 50%, respectively) and resolved on a 1% agarose gel. The RNAs were then blotted onto a nylon membrane (GeneScreen Plus; Perkin Elmer). After UV crosslinking of RNA to the membrane, the blots were prehybridized for 2 h at 42°C in Ultrahyb hybridization buffer (Ambion). A minus-strand oligodeoxyribonucleotide probe with a sequence corresponding to the EV71 positive-strand sequence from nt 105 to nt 133 was end-labeled with [γ-^32^P]ATP and used to probe the RNAs on the nylon membrane by overnight hybridization. The blots were washed twice, each time for 15 min at 25°C with 2×SSC (1×SSC is 0.15 M NaCl plus 0.015 M sodium citrate) and 0.1% SDS, and then exposed for 24 h on a storage phosphor screen. The signals were detected on a Molecular Dynamics Typhoon PhosphorImager scanner and analyzed by ImageQuant software.

### Fluorescence microscopy

RD cells grown on glass cover slips were infected with EV71 at an MOI of 40. At 4, 6, and 8 h post-infection, the culture media were removed, and the cells washed three times with PBS. The cells on the coverslip were fixed with 3.7% (wt/vol) formaldehyde at room temperature for 20 min. After being washed three times with PBS, cells on the coverslip were permeabilized with 0.5% Triton X-100 at room temperature for 5 min and washed again three times with PBS. For AUF1 and EV71 3A immunostaining, the samples were blocked in solution (PBS, containing 5% bovine serum albumin [BSA]) for 60 min at room temperature and then incubated with anti-AUF1 (11,000) or anti-3A antibody (kindly provided by Professor Jim-Tong Horng at Chang Gung University) (1200) for 1.5 h at room temperature and washed three times with PBS. The samples were then reacted with FITC (fluorescein isothiocyanate)-conjugated goat anti-rabbit IgG or rhodamine (tetramethyl rhodamine isothiocyanate [TRITC])-conjugated goat anti-rat IgG (Jackson ImmunoResearch Laboratories, Inc.) for 1 h at room temperature. After being washed with PBS, the samples were treated with DAPI for 15 min at room temperature and washed again with PBS three times. Finally, coverslips with adhered cells were placed on a glass slide and sealed with transparent nail polish. Images were captured by confocal laser scanning microscopy (ZEISS LSM510 META).

### Statistics

Data were compared using the unpaired two-tailed *t* test. *P*<0.05 was considered significant, symbolized by *P<0.05; **P<0.01; ***P<0.001; N.S., not significant.

## Results

### AUF1 associates with viral RNA in EV71-infected cells

**Figure 1:**
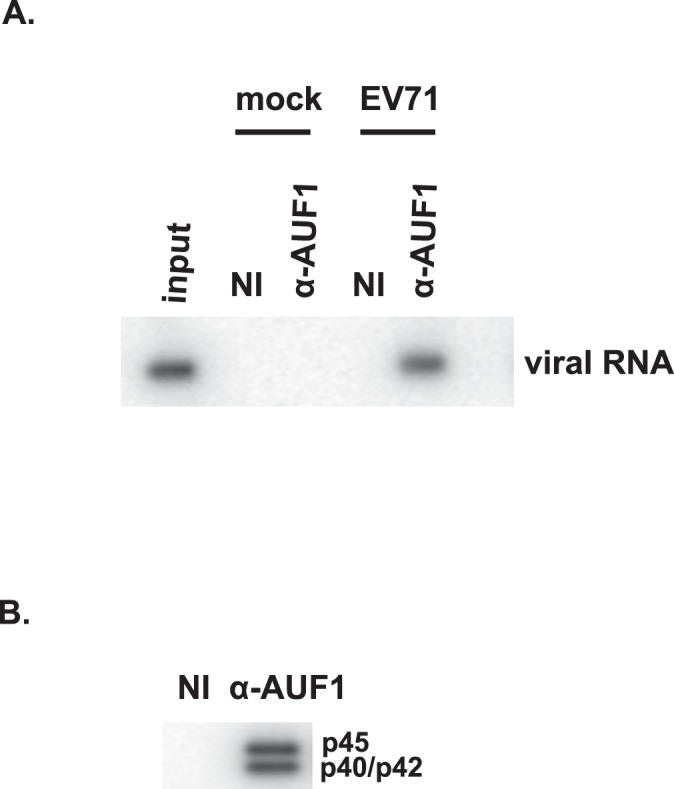
AUF1 associates with EV71 RNA in infected cells. (**A**) Lysates of mock- or EV71-infected SF268 cells were prepared and analyzed by ribonucleoprotein immunoprecipitation (RIP) with non-immune serum or AUF1 anti-serum. Both input and immunoprecipitated materials were analyzed by Northern blotting for EV71 RNA. NI, RIP with non-immune serum. (**B**) Aliquots of immunoprecipitated materials were analyzed by Western blot to verify anti-AUF1–dependent recovery of AUF1. The AUF1 isoforms are indicated.

Semler and colleagues reported that AUF1 associates with the IRES of enterovirus and human rhinovirus and negatively regulates virus infection [26], [39]. To assess whether AUF1 associates with EV71 viral RNA in infected cells, RNP immunoprecipitation and Northern blotting were performed. SF268 cells were infected with EV71 at an MOI of 40 and cell lysates were prepared 8 h post-infection. RNA-protein complexes were immunoprecipitated using non-immune rabbit serum or antiserum specific to AUF1. RNA was isolated from immunoprecipitates and then analyzed by Northern blotting with a radiolabeled probe specific to EV71 RNA. As shown in Figure 1A, immunoprecipitation with AUF1 antibody coprecipitated EV71 RNA while non-immune serum did not. RNA from mock-infected cell also did not present a signal, indicating that the band was not a cellular AUF1 target RNA that might cross-hybridize with the probe. Figure 1B demonstrates that the AUF1 antibody precipitated the indicated AUF1 isoforms; the p40/p42 AUF1 isoforms comigrate on gels but are easily separated from the p45 isoform. The p37 isoform is the least abundant [40] and was not detected in any of the Western blots. As expected, the non-immune serum did not precipitate any detectable AUF1. These results strongly suggest that AUF1 associates with EV71 RNA in cells.

### AUF1 associates with the EV71 5′UTR via stem loop II

**Figure 2:**
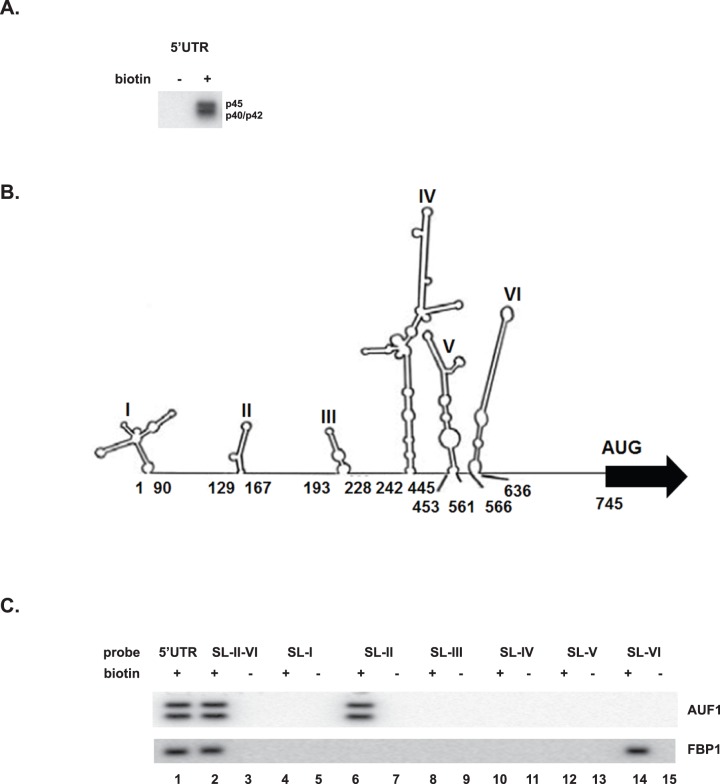
AUF1 interacts with the EV71 5′UTR. (**A**) RNA-protein pull-down experiments were performed to examine the interaction between AUF1 and the EV71 5′UTR. The EV71 5′UTR was synthesized in vitro in the presence of biotin-UTP. Biotinylated RNA was incubated with SF268 cell lysate (200 µg proteins) for 15 min at 30°C. Unlabeled RNA was added to parallel reactions as a negative control. Streptavidin-linked beads were used to pull-down biotin-labeled EV71 5′UTR and its associated cellular proteins. The beads were washed and then resuspended in SDS-PAGE loading buffer to dissociate proteins from RNA. Samples were boiled and analyzed by Western blot using anti-AUF1 antibody. (**B**) Secondary structures within the 5′UTR were predicted by MFold. Numbers below each stem-loop indicate 5′UTR nucleotides encompassing the respective stem-loop. RNA substrates used for protein-RNA pull-down experiments contained the indicated stem-loop and the flanking region immediately 5′ to it. (**C**) Identification of AUF1 interaction site(s) within the EV71 5′UTR. Biotinylated RNAs containing the indicated stem-loops were synthesized; control RNAs lacked biotin. SL-VI extends to nt 745, so it contains linker sequence between the IRES and the AUG. RNA-protein pull-downs and Western blot analysis were carried out as described for panel (A). The blot was stripped and re-probed with anti-FBP1 antibody as a positive control for RNA-binding activity.

Binding of AUF1 to enterovirus RNA was first described by Lenarcic et al. [41]. As noted above, Semler and colleagues showed that AUF1 associates with the IRES of enterovirus and human rhinovirus. The EV71 5′UTR plays roles in viral translation and replication. To determine whether AUF1 interacts with the EV71 5′UTR, the full-length EV71 5′UTR was synthesized in vitro with biotin-UTP. The RNA was mixed with 200 µg of proteins from SF268 cell extract. Streptavidin beads were used to capture biotinylated EV71 5′UTR bound to cellular proteins. Western blotting was carried out to detect AUF1. Figure 2A shows that the indicated AUF1 isoforms associated with the EV71 5′UTR. No pull-down of AUF1 was observed when unlabeled 5′UTR was used.

The 5′UTR of EV71 contains both a cloverleaf structure (nt 1 to 90) and an IRES (nt 91 to 745) (Fig. 2B), as predicted by MFold [14], [42]. To identify the RNA segment(s) in the EV71 5′UTR with which AUF1 associates, biotinylated RNAs corresponding to the various stem loops were synthesized by in vitro transcription. (The numbers in Fig. 2B are the nucleotide numbers from the 5′UTR corresponding to each respective stem-loop RNA.) These were used in pull-down assays with cell extracts. As shown in Figure 2C, the p45 and p40/p42 AUF1 isoforms interacted with the full-length 5′UTR (lane 1) and they interacted with RNA containing SL-II – SL-V. However, AUF1 did not interact with the cloverleaf structure (nt 1 to 90; SL-I) of the 5′UTR (lane 4), which is required for viral RNA replication. In the presence of RNAs containing a single stem-loop of the IRES, AUF1 interacted only with SL-II (lane 6). Thus, RNA containing SL-II appears to be necessary and sufficient for interaction of AUF1 with the 5′UTR. The blot was stripped and re-probed with anti-FBP1 as a positive control for the assay. FBP1 interacts with nt 637–745 of the 5′UTR (i.e., the linker region between SL-VI and the AUG) [13]. As expected, FBP1 associated with RNAs containing the linker between SL-VI and the AUG (Fig. 2C).

### Knockdown of AUF1 promotes viral protein synthesis

**Figure 3:**
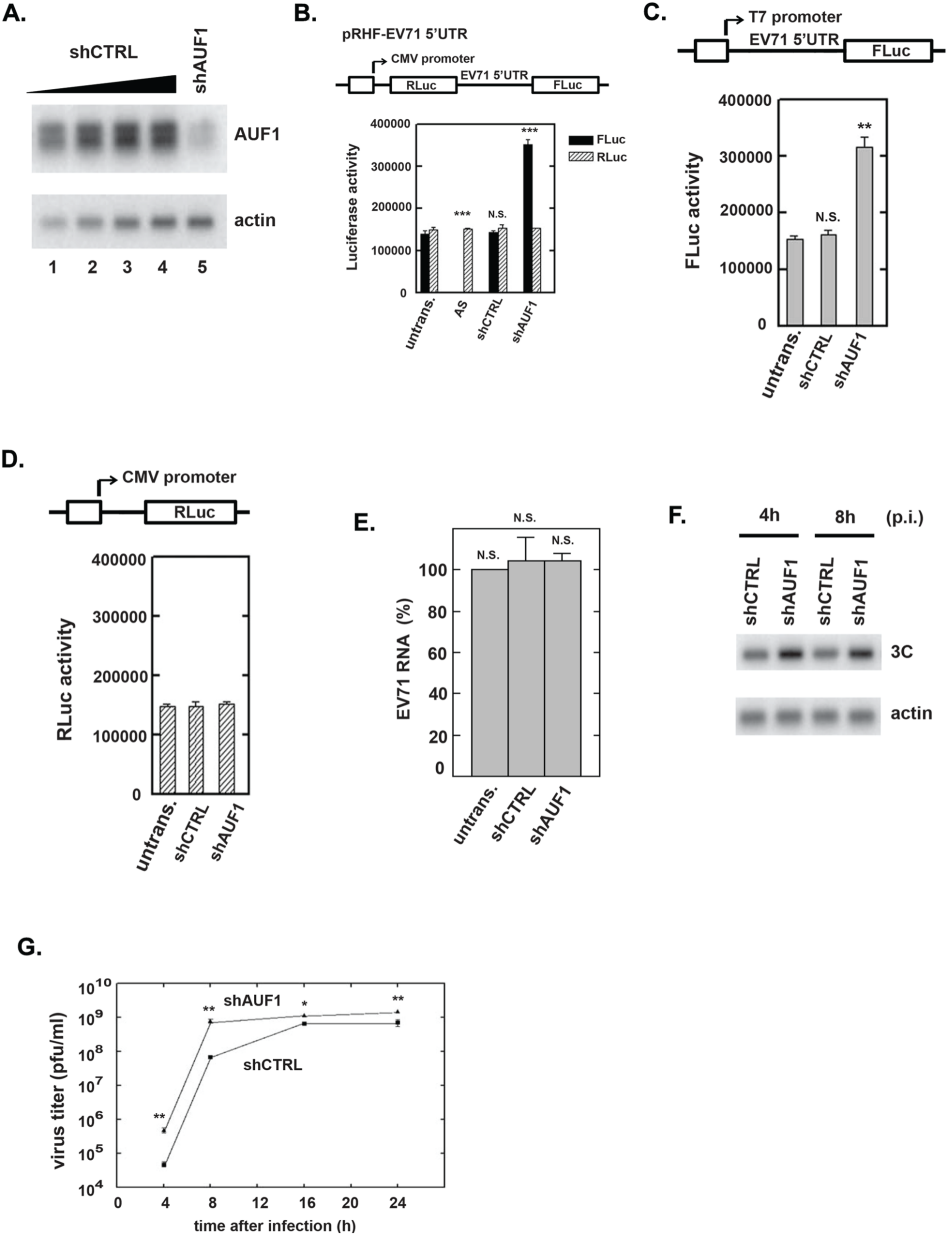
Effect of AUF1 on EV71 IRES activity and viral replication. (**A**) SF268 cells were transfected with plasmids expressing shAUF1 or shCTRL. After 2 days, extracts were prepared for Western blot analyses of AUF1 and β-actin (loading control). A 2-fold dilution series of extract from cells expressing shCTRL (lanes 1 to 4) permitted estimates of AUF1 knockdown efficiency (lane 5). (**B**) Effect of AUF1 knockdown on EV71 IRES activity. The diagram depicts the bicistronic reporter plasmid used to synthesize RNA for transfections and dual-luciferase assays. A control plasmid contained the IRES in the antisense (AS) orientation. SF268 cells were transfected with plasmids expressing shCTRL, shAUF1, or no shRNA (untrans.). Two days after transfection, RLuc-EV71-5′UTR-FLuc RNA was transfected into the cells. Luciferase activity was measured 2 days later. Mean values and standard deviations from three independent experiments are shown in the bar graph. (**C**) Schematic diagram of the IRES-dependent, monocistronic reporter for RNA synthesis. Monocistronic RNA containing the EV71 IRES and FLuc was transfected into cells expressing shCTRL, shAUF1, or no shRNA (untrans.). FLuc activity was measured as described in panel (B). Mean values and standard deviations from three independent experiments are shown in the bar graph. (**D**) Schematic diagram of the cap-dependent, monocistronic reporter. SF268 cells expressing shCTRL, shAUF1, or no shRNA (untrans.) were transfected with cap-dependent reporter RNA. RLuc activity in cell lysates was analyzed two days later. Mean values and standard deviations from three independent experiments are shown in the bar graph. (**E**) Effect of AUF1 knockdown on EV71 RNA levels. Cells were transfected with plasmids expressing shCTRL or shAUF1 or were left untransfected (untrans.). Two days later, cells were infected with EV71 at an MOI of 40 for 8 h. Total RNA was extracted and viral positive-strand RNA levels were determined by qRT-PCR. Mean values and standard deviations from three independent experiments are shown. (**F**) Effect of AUF1 knockdown on EV71 3C protease levels. SF268 cells were transfected with plasmids expressing shAUF1 or shCTRL. Cells were mock infected or infected with EV71 2 days after transfection. Cell lysates were analyzed by Western blotting with anti-3C antibody. Actin served as a loading control. (**G**) Replication of EV71 in AUF1-depleted cells. SF268 cells expressing shAUF1 or shCTRL were infected with EV71 at an MOI of 40 and incubated at 37°C. Medium was harvested 4 and 8 h post-infection (p.i.) and assayed for infectious virus by plaque formation with Vero cells. Mean values and standard deviations from three independent experiments are shown.

AUF1 was mapped to interact within the regions of the IRES reported to participate in translation of viral proteins. To examine the effect of AUF1 on IRES-dependent translation of EV71, expression of AUF1 was reduced by transfection of SF268 cells with a plasmid expressing shRNA against all AUF1 isoforms, shAUF1. (Due to the exon-exon structures of the AUF1 mRNAs, isoform-specific knockdown has not been feasible.) Cells were transfected in parallel with a plasmid expressing control shRNA, shCTRL. Knockdown efficiency was monitored by comparison of serial dilutions of lysate from cells expressing shCTRL and lysate from cells expressing shAUF1. The Western blot indicated AUF1 was reduced >90% (Fig. 3A, compare lane 5 to lane 1). A bicistronic reporter plasmid, pRHF-EV71-5′UTR, was used as a template for synthesis of RLuc-EV71-5′UTR-FLuc RNA. The bicistronic plasmid contains the EV71 5′UTR flanked by the RLuc and FLuc open reading frames (Fig. 3B). Translation of RLuc is cap-dependent; translation of the second cistron, FLuc, is IRES-dependent since translation of FLuc does not occur if the IRES is inserted in the antisense orientation (Fig. 3B, bars labeled “AS”) [16]. Forty-eight hours after transfection of bicistronic RNA, RLuc and FLuc activities were measured using a dual-luciferase reporter assay. Compared to untransfected cells and cells expressing shCTRL, AUF1 knockdown increased IRES-dependent translation (i.e., FLuc) more than twofold (Fig. 3B, solid bars). A comparison of the RLuc activities in control and AUF1-depleted cells indicated that AUF1 knockdown had no effect on cap-dependent translation (Fig. 3B, hatched bars). These results were confirmed using assays of monocistronic reporter RNAs. Depletion of AUF1 promoted IRES-dependent translation (Fig. 3C) but did not affect cap-dependent translation (Fig. 3D). These results indicated that AUF1 acts as a negative regulator of the EV71 IRES.

Effects of AUF1 knockdown on viral RNA synthesis, translation, and titer were examined next. Two days post-transfection of cells with plasmids expressing shCTRL or shAUF1, cells were cultured with 2 µg/ml actinomycin D and infected with EV71 at an MOI of 40. Viral positive-strand RNA levels were examined by real time RT-PCR 8 h post infection. As shown in Figure 3E, AUF1 knockdown had no effect on viral RNA levels. In parallel transfections of shRNA expression plasmids, EV71-infected cells were lysed and Western blotting was performed using antibody against EV71 3C. As shown in Figure 3F, expression of the viral 3C protein was increased upon AUF1 knockdown. Finally, cells transfected with shRNA-expression plasmids were infected with EV71 at an MOI of 40 and viral titers were determined by plaque formation with Vero cells at various times thereafter. Figure 3G shows that virus yield from AUF1-depleted cells increased tenfold at 4 and 8 h post-infection. However, the increase was less pronounced at the 16- and 24-h time points. This indicates that AUF1 negatively regulates EV71 replication, at least at early times post-infection.

### EV71 infection increases cytoplasmic abundance of AUF1

**Figure 4:**
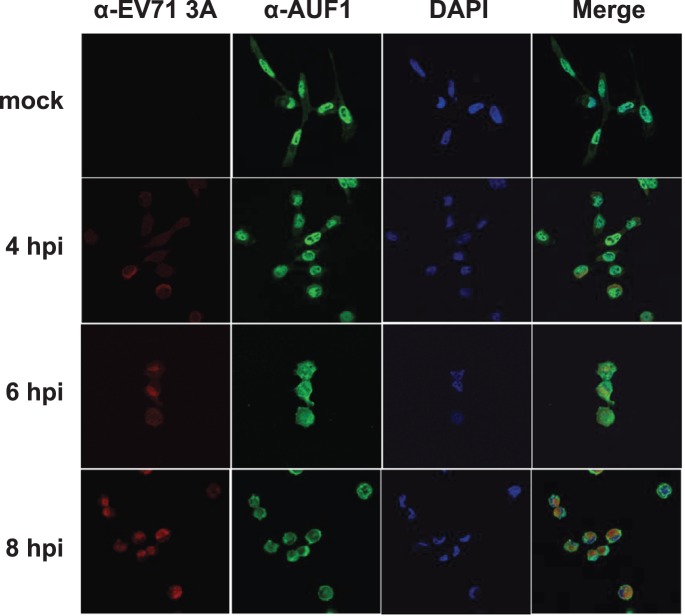
EV71 infection leads to cytoplasmic accumulation of AUF1. RD cells were mock infected or infected with EV71 at an MOI of 40. At 4, 6, and 8-conjugated, goat anti-rabbit IgG or tetramethyl rhodamine isothiocyanate-conjugated, goat anti-rat IgG was used as a secondary antibody. DAPI was used to stain nuclei. Images were captured by confocal laser scanning microscopy.

Although AUF1 presents significant accumulation in the nucleus, the isoforms shuttle between the nucleus and the cytoplasm. Since EV71 replication occurs in the cytoplasm, the effects of infection on the subcellular distribution of AUF1 were examined. Figure 4 shows immunofluorescent staining of AUF1 in EV71-infected RD cells; DAPI was used to stain nuclei. While AUF1 was predominantly nuclear 4 h post-infection, it was distributed evenly in the nucleus and cytoplasm 6 h post-infection, and was almost exclusively in the cytoplasm 8 h post-infection. As expected, infected cells expressed the EV71 3A protein in the cytoplasm (Fig. 4). In comparison, in mock-infected cells, AUF1 was predominantly nuclear.

### AUF1 can compete with hnRNP A1 for IRES association

**Figure 5:**
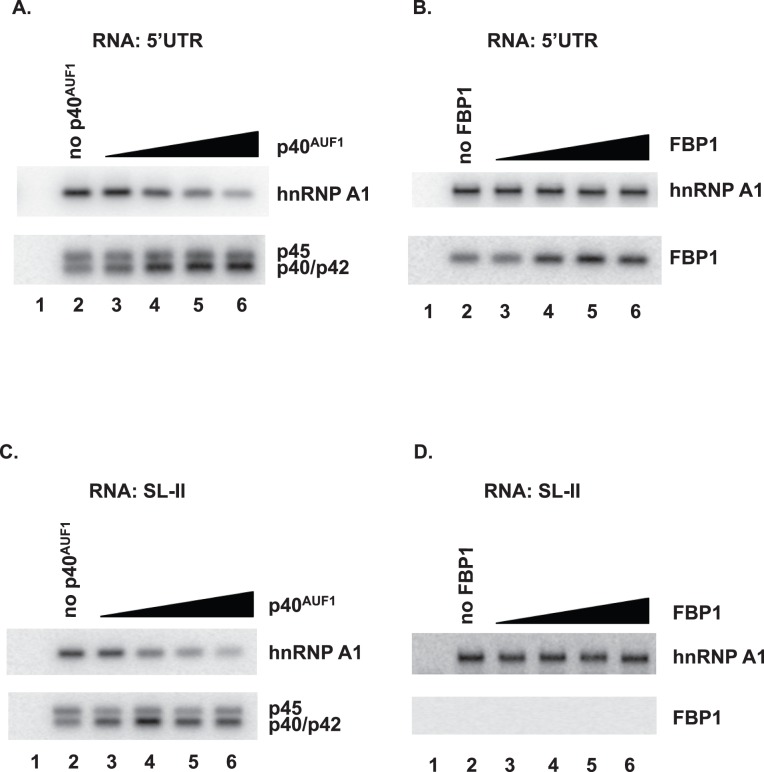
AUF1 can compete with hnRNP A1 for association with the IRES. (**A**) The pull-down assay was performed with SF268 lysate and biotin-labeled EV71 5′UTR as described for Figure 2. Additionally, increasing amounts of purified recombinant p40^AUF1^ were added to reaction mixtures to examine the effects on hnRNP A1 binding to biotin-labeled EV71 5′UTR. Eluted proteins were analyzed by Western blot using anti-hnRNP A1 antibody (upper panel) or AUF1 antibody (lower panel). Lane 1: non-biotinylated RNA was added to the reaction as a negative control. Lanes 3–6: 0.2, 0.5, 1, and 1.5 µg of purified p40^AUF1^ was added to reactions, respectively. (**B**) The pull-down assay was performed as described in panel (A) except increasing amounts of purified recombinant FBP1 were added to the reaction mixtures. (**C**) Biotin-labeled SL-II was used in the pull-down assay to examine the effect of increasing amounts of p40^AUF1^ on hnRNP A1–SL-II interactions. (**D**) Biotin-labeled SL-II and increasing amounts of FBP1 were used in the pull-down assay to assess the effect of FBP1 on hnRNP A1–SL-II interactions.

One mechanism that might account for how AUF1 could reduce IRES activity is that it competes with positive-acting ITAFs for binding to the EV71 5′UTR, thereby reducing their access to the IRES. Our previous work demonstrated that hnRNP A1 associates with SL-II within the EV71 5′UTR and promotes viral translation and replication [16] [43]. AUF1 also associates with SL-II (Fig. 2C). AUF1 and hnRNP A1 are homologous in amino acid sequence and functional domains, particularly in the two RRMs (RNA recognition motifs) and C-terminal glycine-rich region (see Supplementary Fig. 1 in [44]). To determine whether AUF1 and hnRNP A1 might compete for binding to the EV71 5′UTR, a protein-RNA pull-down and competition assay was performed. Biotinylated 5′UTR and 200 µg of protein from cell lysates were mixed in reactions that included increasing amounts of purified recombinant His_6_-p40^AUF1^. This isoform was chosen since it is predominantly cytoplasmic [45], and it is expressed in SF268 cells. Binding was measured by protein-RNA pull-down followed by Western blotting, similar to Figure 2. As shown in Figure 5A, the amount of hnRNP A1 that was RNA-associated decreased with increasing amounts of p40^AUF1^ added; binding by hnRNP A1 was reduced 73% with the maximum p40^AUF1^ added. As expected, increasing p40^AUF1^ in reactions led to its increased association with RNA. As a control for specificity, effects of added FBP1 on binding by hnRNP A1 were examined (Fig. 5B). The amount of hnRNP A1 pulled-down by biotin-5′UTR remained constant despite addition of increasing amounts of FBP1. This is because FBP1 binds the linker region (nt 637–745) of the 5′UTR, while hnRNP A1 binds SL-II [13]. To determine if AUF1 and hnRNP A1 compete for 5′UTR binding via SL-II, biotin-labeled SL-II was used in the RNA pull-down assay. As shown in Figure 5C, the amount of hnRNP A1 associated with SL-II decreased with increasing amounts of p40^AUF1^ added; binding by hnRNP A1 was decreased by 80% with the maximum amount of p40^AUF1^ added. By contrast, the amount of hnRNP A1 associated with SL-II remained constant with increasing amounts of FBP1 added; SL-II did not pull-down FBP1, since it does not bind SL-II, as noted above (Fig. 5D). Taken together, these results suggest reciprocal binding of SL-II by AUF1 and hnRNP A1.

**Figure 6:**
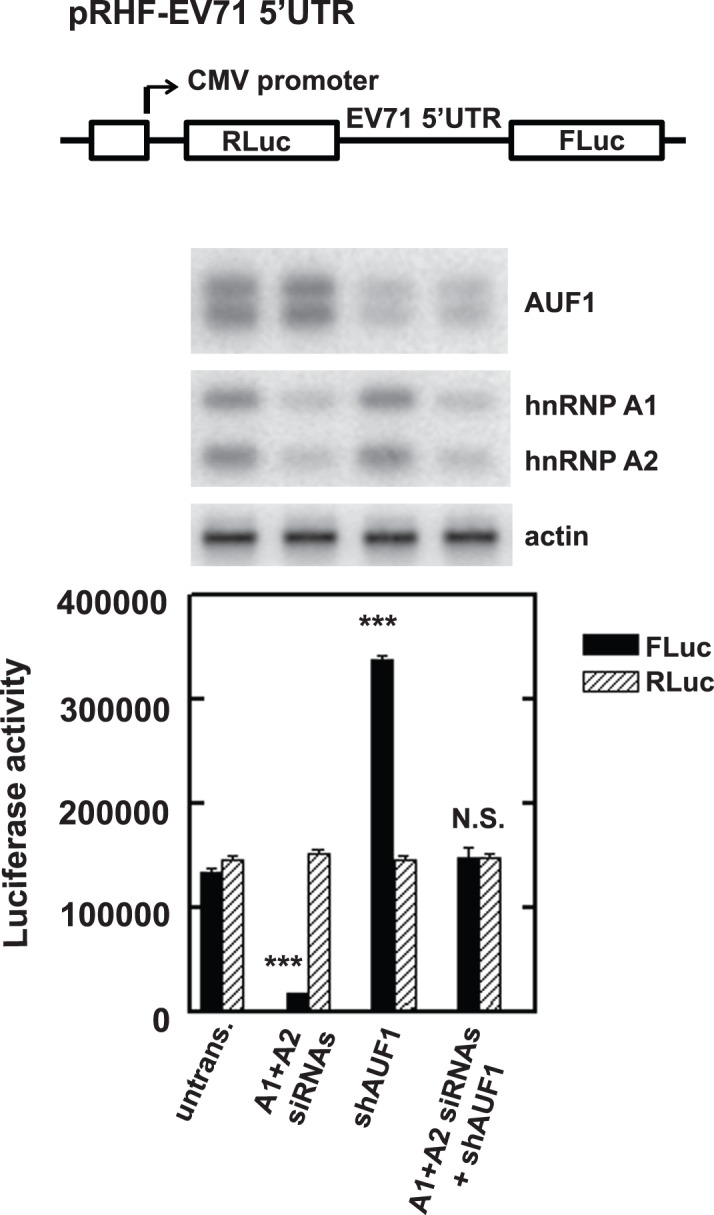
Effect of depleting AUF1 and/or hnRNP A1/A2 on EV71 IRES activity. SF268 cells were transfected with the indicated siRNAs and/or shRNA plasmids for two days. RLuc-EV71-5′UTR-FLuc RNA was then transfected into cells. Levels of hnRNP A1/A2 and AUF1 were analyzed by Western blot and luciferase activity was measured 2 days later. Mean values and standard deviations from three independent experiments are shown in the bar graph. Each lane of the Western blot corresponds to the siRNA/shRNA transfections indicated at the bottom of the bar graph.

To assess the effects of these proteins on EV71 IRES activity, AUF1 and hnRNP A1 were depleted in SF268 cells and the effects on IRES activity were examined as described in Figure 3. Our previous work demonstrated that hnRNP A2 can also interact with the EV71 IRES [16]. Thus knockdown of both hnRNPs A1 and A2 was required. (See Western blots in Fig. 6). Knockdown of hnRNPs A1 and A2 reduced IRES activity (i.e., FLuc) dramatically, while knockdown of AUF1 enhanced IRES activity (Fig. 6). Thus, their effects on IRES activity are reciprocal, just like their IRES binding activity is. Combined knockdowns of hnRNP A1/A2 and AUF1 had no effect on IRES activity. This result suggests that the IRES has an intrinsic activity that might be tuned up or down by hnRNP A1/A2 and AUF1, respectively.

## Discussion

This work shows that AUF1 interacts with EV71 RNA in cells (Fig. 1) and its highly structured 5′UTR in vitro (Fig. 2). AUF1 also acts as an ITAF that negatively regulates both IRES-dependent translation and viral replication (Fig. 3). However, AUF1 appears not to affect viral RNA synthesis (Fig. 3).

AUF1 and hnRNP A1 are in the same hnRNP family, and highly homologous, with two RRMs and a glycine-rich C-terminus [44]. Both proteins interact with SL-II in the EV71 5′UTR. HnRNP A1 is a positive-acting ITAF that stimulates IRES-dependent translation of EV71 [16]. Our results showed that AUF1 can compete with hnRNP A1 for association with SL-II in the 5′UTR (Fig. 5), suggesting that interaction of AUF1 with SL-II may block hnRNP A1 binding and inhibit viral translation. In collaboration with Tolbert and colleagues (Case Western Reserve University), we showed that SL-II forms a 5-nt bulge, capped by a 6-nt hairpin structure that is conserved among enterovirus IRESs. The bulge and hairpin loops act cooperatively to allow formation of an hnRNP A1–SL-II complex. Mutations that weaken cooperative binding result in a significant decrease in IRES-dependent translation and impaired EV71 replication [43]. Future work will examine whether AUF1 also binds to the bulge and hairpin loops of SL-II and whether the mutations in the bulge and hairpin that weaken hnRNP A1 binding also affect its interaction with AUF1.

We demonstrated that AUF1 interacts with the EV71 5′UTR through SL-II. Semler and colleagues reported that AUF1 directly interacts with SL-IV of the poliovirus 5′UTR and inhibits viral translation [26]. We speculate that one mechanism by which AUF1 could inhibit IRES-dependent translation of EV71 (i.e., by competitive binding with hnRNP A1) may be different from that of poliovirus. It would not be surprising that the same protein may act differently for different enteroviruses. For example, AUF1 exerts an antiviral property by targeting coxsackievirus B3 RNA for degradation via binding the 3′UTR [27]. Recently Cathcart and Semler [46] demonstrated that AUF1 does not inhibit all picornavirus infections. Replication of encephalomyocarditis virus (EMCV), a cardiovirus in the picornavirus family, is not AUF1-dependent. AUF1 re-localizes to the cytoplasm after EMCV infection but is not cleaved as it is during enterovirus infections.

AUF1 is involved in DNA and RNA viral infections. Semler and colleagues reported that AUF1 interacts with SL-IV of the poliovirus 5′UTR to reduce translation and replication. AUF1 negatively regulates replication of three related picornaviruses – polio-, human rhino-, and coxsackievirus – in mammalian cells. These viruses appear to have overcome this inhibitory effect, in part, by promoting the relocalization of AUF1 and by proteolytic cleavage of AUF1 [26], [39]. Wong and colleagues reported that AUF1 is relocalized and cleaved at the N-terminus during coxsackievirus B3 infection. However, as noted above, AUF1 can act as an antiviral factor by targeting the viral RNA for degradation [27]. In contrast to its negative role in poliovirus translation, AUF1 positively regulates the translation of another positive-strand RNA virus, hepatitis C virus (HCV) [24]. Similarly, during HIV infection, AUF1 may play a role in HIV Gag and Env synthesis either through affecting RNA stability or by export or alteration of the RNP complex involved in export [31]. In addition to roles in RNA virus infection, AUF1 interacts with the Epstein-Barr virus F promoter, the latency C promoter, and the EBER1 noncoding RNAs [25], [47], [48]. These findings, along with work described in this paper, further highlight the diverse functions of AUF1 in virus replication.
